# The association between core functions of algorithmic management and work-related health problems: indications for effects beyond well-known psychosocial stressors

**DOI:** 10.3389/fpubh.2026.1886482

**Published:** 2026-08-26

**Authors:** Andreas Müller, Ivaneta Danailova, Michael Ertel, Harald Gündel, Meike Heming, Anne Kemter, Louisa Scheepers, Carsten Röttgen

**Affiliations:** 1 Institute of Psychology, Work and Organizational Psychology, University of Duisburg-Essen, Universitätsstr, Essen, Germany; 2 Federal Institute for Occupational Safety and Health, Berlin, Germany; 3 Clinic for Psychosomatic Medicine and Psychotherapy, University of Ulm, Ulm, Germany; 4 Institute of Occupational, Social and Environmental Medicine, Centre for Health and Society, Medical Faculty and University Hospital Düsseldorf, Heinrich Heine University, Düsseldorf, Germany; 5 Federal Institute for Occupational Safety and Health, Dresden, Germany

**Keywords:** algorithmic management, digitalisation, employee well-being, risk assessment, work design

## Abstract

**Introduction:**

Algorithmic management (AM) marks a transformation in work organization as managerial decisions increasingly rely on data-driven algorithms. While research suggests that AM may affect employee health, empirical evidence remains fragmented. This study examines whether associations between AM and work-related health impairments generalize across occupations.

**Methods:**

Data from the cross-sectional Flash Eurobarometer “OSH Pulse” (*N* = 27,242 workers) were analyzed using cross-classified mixed-effects logistic regression. Exposure to AM—algorithmic monitoring, task allocation, and performance evaluation—was examined in relation to self-reported health problems (stress, depression or anxiety; musculoskeletal problems; headaches or eyestrain; accidents or injuries; overall fatigue), controlling for demographic variables and psychosocial work stressors.

**Results:**

Findings show that with each additional AM function the likelihood of reported health problems increased modestly but constantly (OR = 1.06–1.11). Among the single AM functions examined, monitoring appeared to have most consistent associations with workers health problems.

**Discussion:**

Findings suggest that AM may represent an additional risk factor for worker health accross different occupations. However, results should be interpreted cautiously due to non-established screening measures and an overrepresentation of tertiary-educated workers.

## Introduction

A recent development in workplace digitalization is Algorithmic Management (AM) (1) “the use of computer-programmed procedures for the coordination of labor input in an organization” (2). AM is characterized by functions such as algorithmic task allocation, scheduling, and performance monitoring—activities previously performed by human managers (3, 4). Its implementation marks a fundamental paradigm shift in work organization, as decision-making and control move from humans to algorithms (5). This is most visible in platform organizations, where digital systems allocate gig work and evaluate performance (6), but similar developments are spreading to traditional sectors including logistics and health care (7).

Drawing on sociomaterial system theory (8) and established work design theories (9), and the Job Demands-Resources Theory (10), conceptual work suggests that AM can affect employee’s health particularly by reducing job resources and increasing quantitative job demands (3, 4). Empirical findings largely support this view, linking AM to lower job autonomy combined with a higher work pace, which subsequently leads to poorer well-being (11, 12). Nevertheless, the empirical foundation supporting this relationship remains both fragmented and methodologically constrained (13).

Given this inconclusive evidence, we examine whether AM effects on employee health generalize across contexts, using data from a representative random probability sample of over 27,000 European workers. Beyond advancing research on AM and workers’ health, the study informs regulatory initiatives advocating stronger integration of AM into occupational health and safety frameworks (14).

## Method

### Design and sample

The study used the data from the Flash Eurobarometer “OSH Pulse—Occupational safety and health in post-pandemic workplaces” (15). The cross-sectional survey was aimed at employees from the age 16 or older who are citizens or residents of the 27 countries of the European Union as well as Norway and Iceland with sufficient language skills of one of the respective languages of the country to answer the questionnaire. It was conducted between April and May 2022 in the form of computer-assisted telephone interviews. The sample design is a random probability design, which minimizes selection bias, leading to samples that are more likely to reflect the targeted working population. Altogether 27,242 employees participated. The data set (ZA8753; Version 2.0.0) is openly available at https://doi.org/10.4232/1.14208.

### Measures

The “OSH Pulse” survey included screening measures on the use of digital technologies at work, psychosocial risk factors in the workplace, work-related health problems, and occupational health and safety management. In the following, we describe the items used for our analyses. For all items, the original coding was retained unless otherwise specified.

*Work-related health problems* were assessed using five items which asked: “In the last 12 months, have you experienced any of the following health problems caused or made worse by your work?” (1) stress, depression, or anxiety; (2) bone, joint, or muscle problems or pain; (3) headaches or eyestrain; (4) accidents or injuries; and (5) overall fatigue. Each item was binary coded (1 = health problem reported, 0 = not mentioned). Two items—infectious diseases and “another health problem”—were excluded from the analyses, as the relation of the first to AM was considered implausible and the latter was regarded as too unspecific.

*AM* was evaluated using three items, asking: “To your knowledge, does the organization where you work use digital devices such as a tablet, smartphone, computer, laptop, app, or sensor to…” (1) supervise or monitor your work and behavior personally, (2) automatically allocate tasks, working time, or shifts to you, and (3) have your performance rated by third parties. The items mirror the main functions of AM—algorithmic monitoring, task allocation, and performance evaluation – and are consistently identified in conceptual frameworks and existing AM measurement instruments as central components of algorithmic management (3, 4, 11, 12). The three items were coded the as: yes = 1, no = 0, do not know = missing. A sum score was created, ranging from 0 to 3, with higher scores indicating more comprehensive AM.

*Psychosocial work stressors* were assessed with five items (yes = 1, no = 0, do not know = missing), assessing (1) time pressure or work overload, (2) violence or verbal abuse from customers, patients, or pupils, (3) harassment or bullying, (4) poor communication or cooperation, and (5) lack of autonomy. These stressors correspond to well-known psychosocial risk factors that have been empirically linked to various adverse health outcomes (16). A sum score was created, ranging from 0 to 5, with higher scores indicating a more severe exposure to the assessed work stressors.

We considered the following *individual level confounders*. With the exception of age, these were recorded as categorical variables and dummy-coded by us: gender (male as reference; dummy variables for female and diverse), age (continuously in years), education (tertiary education as reference; dummy variables for primary, secondary, still in full-time education, and never in full-time education), employment status (permanent contract as reference; dummy variables for self-employment and temporary employment), fulltime employment (1 = fulltime, 0 = parttime). Do not know or refusal answers in all variables were recoded as missing. Additionally, analyses applied the weighting score provided by the OSH Pulse study to align the sample with official population statistics on gender, age, and region (15).

In addition, we included the two nominal *level 2 confounders* country (one category each for the 27 countries of the European Union, as well as Norway and Iceland) and 10 economic sectors (with the statistical classification of economic activities (NACE: 1. administrative and support services and public administration; 2. agriculture; 3. mining and water, gas, electricity supplies; 4. construction; 5. manufacturing; 6. commerce, transport and logistics, accommodation and food services; 7. ICT, finance, professional and technical services; 8. education; 9. health and social care activities; 10. other service activities).

### Analyses

A series of stepwise cross-classified mixed-effects logistic regression analyses using R (17) was performed. For hypothesis testing, missing data were handled using multiple imputation with 20 imputed datasets, with 10 iterations per dataset implemented with the mice package (18). For imputation, we used predictive mean matching for continuous variables, logistic regression for binary nominal variables, and polytomous regression for multinominal variables. For each of the five health problems as outcome measure, we compared four models to evaluate model fit and sensitivity of effects: (1) A *Null-Model* including only the random effects of level 2 variables country and sector, (2) an *AM-Model* including the effects of AM only, (3) a *Full-Model* including the additional effects of the individual level confounders beyond AM, and (4) a *Full Random Slopes (RS)-Model* additionally allowing the slopes of AM effects to vary across level-2 variables. This resulted in a total of 20 estimated models (5 health outcomes × 4 models). Additionally, we assessed for each of the health problems a Full-Model that simultaneously estimated the effects of the three single AM-functions. Models were specified as cross-classified rather than strictly hierarchical, since individuals simultaneously belong to two non-nested, mutually independent group structures (country and sector). Parameters were estimated separately for each of the 20 imputed datasets and combined according to recommendations of Rubin (19). Results were expressed as odds ratios (ORs). To quantify explained variance, we calculated marginal and conditional pseudo-R^2^ following Nakagawa and Schielzeth (20), Model comparisons across imputed datasets were based on the average deviance difference (−2 log-likelihood) between nested models across the imputed datasets.

## Results

Descriptive statistics are displayed in Table 1. Nearly half of the sample reported no AM functions in their workplace. About one third indicated the presence of at least one AM function, while approximately 8% reported all three functions. Time pressure was by far the most reported work stressor, affecting almost half of the participants, whereas bullying was the least frequently reported stressor. Regarding health problems, fatigue was most frequently reported (by more than one third of participants), followed by headache and eyestrain.

**Table 1:** Descriptive statistics.

Variable	Category	Total sample *N* = 27,242	
		Freq.	Percent
Age	16–24 years	2,116	7.8
	25–39 years	9,093	33.4
	40–54 years	10,652	39.1
	55 years and older	5,381	19.8
Gender	Male	14,608	53.6
	Female	12,438	45.7
	Diverse	196	0.7
Education	Primary education	369	1.4
	Secondary education	8,618	31.6
	Tertiary education	16,836	61.8
	Still in full-time education	941	3.5
	Never in full-time education	174	0.6
	Do not know	285	1.0
	Refusal	19	0.1
Employment status	Self-employed	4,289	15.7
	Permanent contract	19,651	72.1
	Temporary contract	3,302	12.1
Fulltime contract	Part time	3,950	14.5
	Fulltime	23,088	84.8
	Do not know	204	0.7
Health problems (last 12 months)	Stress, depression, anxiety	7,697	28.3
	Musculoskeletal problems	8,086	29.7
	Headaches, eyestrain	9,811	36.0
	Overall fatigue	10,395	38.2
	Accident or injuries	1,477	5.4
Work stressors	time pressure	12,284	45.1
	violence or verbal abuse	4,009	14.7
	harassment or bullying	1,893	6.9
	poor cooperation	7,242	26.6
	lack of autonomy	4,466	16.4
Algorithmic management (AM)	Monitoring	7,748	28,4
	Task allocation	8,311	30.5
	Performance evaluation	8,351	30.7
	no AM reported	11,241	41.3
	one AM practice reported	7,635	28.0
	two AM practices reported	4,597	16.9
	three AM practices reported	2,184	8.0
	Do not know	1,585	5.8

**Figure 1:**
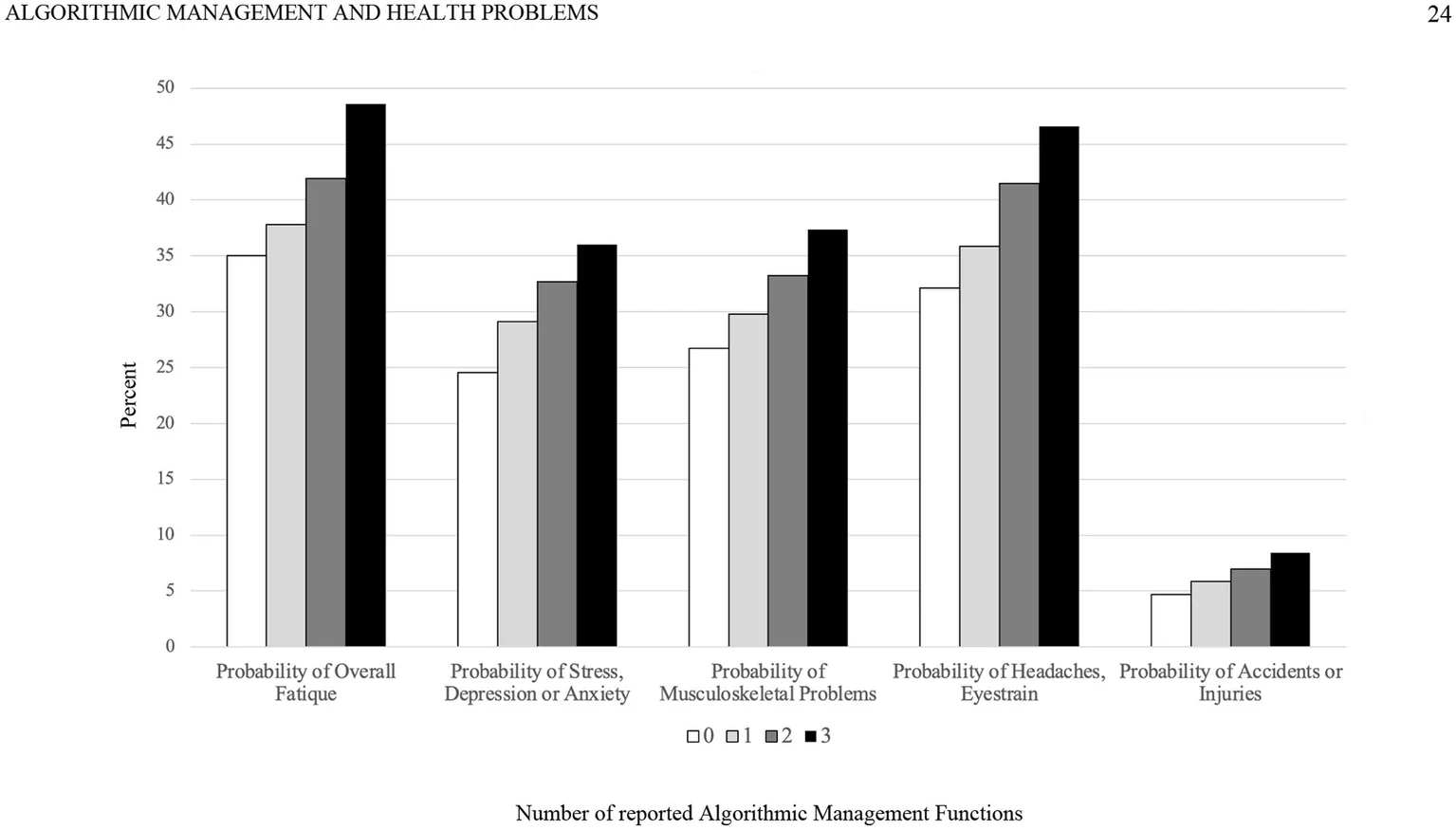
Probability of health problems in relation to the number of algorithmic management functions (*N* = 27.139).

Figure 1 shows a graded increase in the prevalence of all investigated health problems with higher numbers of AM functions at the workplace. Compared with workers exposed to no AM functions, those exposed to three AM functions had a higher probability of overall fatigue, mental health symptoms, musculoskeletal problems, headaches or eyestrain, and occupational accidents or injuries. The observed dose–response pattern is consistent with an association between greater exposure to AM and poorer health outcomes.

A total of 2,642 participants (9.6%) had missing values, i.e., they selected the answer “do not know” or refused to answer for one or more items of the included variables. Little’s MCAR test indicated that the missing data were not at random [*χ*^2^(2543) = 3,591, *p* < 0.001]. Therefore, we created 20 datasets using multiple imputation of missing values to analyze the data with the complete sample.

Table 2 provides an overview about the fit of all tested models as well as the explained variance in the outcomes. Across all five health outcomes, model fit improved substantially and consistently from the Null-model through the AM-model up to the Full model, as indicated by decreasing AIC and BIC and significant *χ*^2^-differences (all *p* < 0.001). The intraclass correlation coefficient (ICC) of the Null-model showed that random intercepts of level 2 variables country and sector alone explained 3.4–7.4% of the total variance in the outcome, which was mainly attributable to country. That is why, in the Full RS-models, for reasons of parsimony we only allowed for a variation of AM-slopes across countries but not across sectors.

**Table 2:** Model-fit.

Model	AIC	BIC	Δ*χ*^2^ (vs. previous)	*p*	*R*^2^ marginal	*R*^2^ conditional	ICC
Outcome: overall fatigue							
Null	35,014.12	35,038.75			0.000	0.066	0.066
AM	34,841.38	34,874.23	174.74 (df = 1)	<0.001	0.008	0.075	0.067
Full	32,614.77	32,746.17	2250.61 (df = 12)	<0.001	0.112	0.182	0.079
Full RS	32,652.67	32,800.50	−33.91 (df = 2)	n.s.	0.117	0.182	0.016
Outcome: stress, depression or anxiety							
Null	31,918.17	31,942.80			0.000	0.036	0.036
AM	31,733.19	31,766.04	186.97 (df = 1)	<0.001	0.010	0.045	0.036
Full	29,025.33	29,156.74	2731.86 (df = 12)	<0.001	0.147	0.172	0.029
Full RS	29,062.81	29,210.63	−33.47 (df = 2)	n.s.	0.150	n/a	0.003
Outcome: musculoskeletal problems							
Null	32,628.09	32,652.73			0.000	0.034	0.034
AM	32,493.96	32,526.81	136.14 (df = 1)	<0.001	0.007	0.041	0.034
Full	31,244.72	31,376.12	1273.23 (df = 12)	<0.001	0.073	0.108	0.038
Full RS	31,284.44	31,432.26	−35.71 (df = 2)	n.s.	0.074	n/a	0.008
Outcome: headaches, eyestrain							
Null	34,862.33	34,886.97			0.000	0.045	0.045
AM	34,638.89	34,671.74	225.44 (df = 1)	<0.001	0.011	0.054	0.044
Full	32,997.67	33,129.07	1665.22 (df = 12)	<0.001	0.092	0.137	0.050
Full RS	33,042.13	33,189.96	−40.46 (df = 2)	n.s.	0.093	0.137	0.010
Outcome: accidents or injuries							
Null	11,267.36	11,292.00			0.000	0.074	0.074
AM	11,214.23	11,247.08	55.13 (df = 1)	<0.001	0.011	0.086	0.076
Full	10,747.93	10,879.33	490.3 (df = 12)	<0.001	0.089	0.144	0.061
Full RS	10,741.84	10,889.67	10.09 (df = 2)	<0.01	0.087	0.147	0.129

AIC, Akaike information criterion; BIC, Bayesian information criterion; OR, odds ratio; AM = Algorithmic Management. RS = random slope of AM-effects across countries. *R*^2^ marginal: variance explained by fixed effects; *R*^2^ conditional: variance explained by fixed and random effects combined; ICC, adjusted intraclass correlation coefficient: proportion of variance that is attributable to country and sector-level random effects after accounting for fixed effects; n/a: conditional *R*^2^ could not be computed, because model showed a degenerate random-effects covariance matrix. All values were averaged across 20 imputed datasets.

Multicollinearity in the Full-Model was assessed separately in each imputed dataset using generalized variance inflation factors (VIF). Maximum VIF values of predictors ranging from 1.13–1.17 across imputations, indicating no multicollinearity among the fixed-effect predictors.

For all outcomes, the AM sum score alone explained a small but significant proportion of variance (marginal R^2^ increasing to 0.007–0.011). The Full model, additionally including confounders, substantially increased explained variance (marginal *R*^2^ = 0.07–0.15). The Full RS-models did not converge for four of the five health outcomes, which indicates insufficient country-level variation in the slopes of AM effects. In contrast, for occupational accidents, the Full RS-model converged and showed improved fit compared to the Full model [Δ*χ*^2^(2) = 10.09, *p* = 0.006]. Moreover, there was a marked increase in the country-level intraclass correlation compared to the Full-model (from 0.061 to 0.129), suggesting meaningful cross-country variation in the effect of AM on accident risk.

The pooled estimates of the Full-model, which consistently showed the best fit, are displayed in Table 3. AM emerged as a consistent and significant predictor over and above demographic variables and well-known work stressors across all models. More comprehensive AM was associated with an increased likelihood of reporting stress, depression or anxiety, musculoskeletal problems, headaches or eyestrain, overall fatigue, and accidents or injuries. With each additional AM-characteristic, the odds of experiencing these health outcomes rise by 6–11%. For occupational accidents or injuries, the Full RS-model indicated that AM was not associated with an increased risk in most countries. Statistically significant associations were found only in Greece (OR = 1.27, 95% CI: 1.05–1.53) and Sweden (OR = 1.51, 95% CI: 1.27–1.81).

**Table 3:** Mixed-effects logistic regression predicting work-related health problems from algorithmic management (AM).

Predictor	Overall fatigue		Stress, depression or anxiety			Musculoskeletal problems			Headaches, eyestrain			Accidents or injuries			
	OR	95% CI	*p*	OR	95% CI	*p*	OR	95% CI	*p*	OR	95% CI	*p*	OR	95% CI	*p*
Intercept	0.02	[0.02, 0.03]	<0.001	0.01	[0.01, 0.01]	<0.001	0.02	[0.01, 0.02]	<0.001	0.03	[0.02, 0.04]	<0.001	0.00	[0.00, 0.01]	<0.001
Weighting score	1.01	[0.97, 1.05]	0.789	0.94	[0.90, 0.98]	0.004	1.04	[1.00, 1.08]	0.065	0.97	[0.94, 1.01]	0.169	1.01	[0.94, 1.08]	0.814
Age	0.99	[0.99, 0.99]	<0.001	0.99	[0.99, 0.99]	<0.001	1.01	[1.01, 1.01]	<0.001	0.99	[0.99, 0.99]	<0.001	0.99	[0.99, 1.00]	<0.001
Gender (female)	1.31	[1.24, 1.39]	<0.001	1.30	[1.22, 1.38]	<0.001	1.35	[1.27, 1.43]	<0.001	1.56	[1.47, 1.65]	<0.001	0.73	[0.65, 0.82]	<0.001
(diverse)	1.00	[0.73, 1.37]	0.992	1.09	[0.78, 1.54]	0.601	1.52	[1.12, 2.06]	0.007	1.22	[0.90, 1.66]	0.197	0.91	[0.51, 1.63]	0.749
Education (primary)	1.39	[1.11, 1.74]	0.004	0.86	[0.67, 1.10]	0.222	1.61	[1.29, 2.01]	<0.001	0.84	[0.67, 1.07]	0.164	1.79	[1.21, 2.65]	0.004
(secondary)	1.01	[0.95, 1.07]	0.849	0.85	[0.79, 0.90]	<0.001	1.37	[1.29, 1.46]	<0.001	0.86	[0.81, 0.92]	<0.001	1.45	[1.29, 1.63]	<0.001
(in education)	0.99	[0.85, 1.16]	0.897	0.95	[0.81, 1.13]	0.587	1.02	[0.86, 1.20]	0.858	1.03	[0.89, 1.20]	0.684	1.35	[1.02, 1.81]	0.039
(never in full time)	1.02	[0.72, 1.45]	0.926	0.95	[0.65, 1.38]	0.789	1.46	[1.04, 2.05]	0.028	0.96	[0.68, 1.36]	0.812	2.03	[1.22, 3.38]	0.006
Employment status (self-employed)	1.05	[0.98, 1.13]	0.191	1.20	[1.11, 1.30]	<0.001	1.13	[1.04, 1.22]	0.003	0.98	[0.91, 1.05]	0.557	1.25	[1.07, 1.45]	0.004
(fixed term)	1.01	[0.93, 1.10]	0.731	0.95	[0.87, 1.04]	0.280	1.05	[0.97, 1.15]	0.243	0.88	[0.81, 0.96]	0.003	1.21	[1.03, 1.43]	0.017
Fulltime	1.12	[1.03, 1.21]	0.007	1.09	[1.00, 1.19]	0.057	0.97	[0.89, 1.05]	0.409	1.11	[1.02, 1.20]	0.013	0.94	[0.81, 1.10]	0.456
Work stressors	1.71	[1.67, 1.75]	<0.001	1.85	[1.80, 1.89]	<0.001	1.44	[1.41, 1.48]	<0.001	1.52	[1.48, 1.55]	<0.001	1.53	[1.46, 1.59]	<0.001
AM	1.06	[1.03, 1.09]	<0.001	1.06	[1.03, 1.09]	<0.001	1.08	[1.05, 1.11]	<0.001	1.11	[1.08, 1.15]	<0.001	1.07	[1.01, 1.13]	0.014

*N* = 27,242. Results represent pooled estimates of 20 imputed data sets. Models included random intercepts for level-2 factors country and sector. Weighting score matched the sample with the official population statistics of the participating countries. Age in years; Gender = Dummy Coded (reference category “male”); Education = Dummy Coded (reference category “tertiary education”); Employment = Dummy coded (reference category “permanent employment”); Fulltime = Dummy coded (reference category “Parttime”); Work Stressors and AM represent sum scores.

Additional analyses using a dataset with listwise deletion of participants with missing data yielded almost identical AM effects; only the AM effects on musculoskeletal problems and accidents differed slightly by +/− 0.01 OR.

Table 4 shows the simultaneous effects of the single AM functions in the Full-model. As the only AM function, algorithmic monitoring was consistently related to a higher risk of health problems across all five outcomes. Algorithmic task allocation and performance rating were only related to a higher probability of two health problems: musculoskeletal problems, headache and eyestrain.

**Table 4:** Mixed-effects logistic regression predicting work-related health problems from single algorithmic management (AM) functions.

Predictor	Overall fatigue		Stress, depression or anxiety			Musculoskeletal problems			Headaches, eyestrain			Accidents or injuries			
	OR	95% CI	*p*	OR	95% CI	*p*	OR	95% CI	*p*	OR	95% CI	*p*	OR	95% CI	*p*
AM monitoring	1.12	[1.05, 1.19]	<0.001	1.11	[1.04, 1.19]	0.002	1.09	[1.02, 1.16]	0.012	1.09	[1.02, 1.16]	0.009	1.14	[1.01, 1.29]	0.032
AM task allocation	1.03	[0.97, 1.09]	0.406	1.02	[0.95, 1.08]	0.636	1.07	[1.01, 1.14]	0.023	1.08	[1.02, 1.15]	0.010	1.04	[0.92, 1.17]	0.564
AM performance evaluation	1.03	[0.97, 1.10]	0.330	1.06	[0.99, 1.13]	0.099	1.09	[1.02, 1.16]	0.008	1.17	[1.10, 1.25]	<0.001	1.04	[0.92, 1.17]	0.539

*N* = 27,242. Results represent pooled estimates of 20 imputed data sets. Models included random intercepts for country and sector. Weighting score matched the sample with the official population statistics of the participating countries. Effects are controlled for age, gender, education, employment status, part time work, work stressors.

## Discussion

Our findings suggest that algorithmic management (AM) is weakly but consistently associated with workers self-reported health problems beyond established psychosocial work stressors. The present results are in accordance with previous studies reporting adverse health effects associated with AM (11, 12, 21, 22). However, this prior evidence has largely been limited either to specific occupational groups, such as logistics workers, or was based on risk assessments provided by HR managers or employee representatives who have only indirect insights into the health situation of workers. By using a population-based sample of workers in Europe, the present study contributes to the generalizability of these findings across a broader workforce.

Our findings suggest that the health-related implications of AM are broadly consistent across sectors and countries. One exception appears to be the association with occupational accidents and injuries, where the strength of the observed associations varied between countries. This heterogeneity may reflect differences in national occupational health and safety regulations that might shape AM design and worker protection. However, further research is needed to better understand the contextual factors underlying these cross-country differences.

Among the AM functions examined, algorithmic monitoring appeared to have the greatest relevance for workers’ health. This finding is plausible, as continuous monitoring constitutes a core component of AM systems, providing the real-time data required for algorithmic task allocation, performance evaluation, and other AM functions (3, 4, 11, 12). Our results are also consistent with evidence beyond the AM literature indicating that workplace surveillance is associated with increased stress and poorer psychosocial well-being. These associations have been attributed to mechanisms such as heightened evaluation anxiety, reduced perceived autonomy, and a persistent sense of being observed, all of which may contribute to adverse health outcomes (23).

The unique independent effects of AM appear modest in magnitude. However, building on prior conceptual work on AM and work design, AM may contribute to employee strain not only directly but also indirectly through changes in health-relevant job characteristics (4). Core AM functions such as monitoring, task allocation, and performance evaluation can intensify external control over workers (5), which may for example reduce job autonomy and increase work pace (11). In addition, AM systems may reduce social support by limiting direct interpersonal interaction and promoting a more instrumentalized form of social exchange (24). Consistent with this interpretation, the pooled correlation between AM and the work stressor score across the 20 imputed datasets in our study was *r* = 0.19, 95% CI [0.18, 0.20], indicating a systematic association between AM exposure and elevated work stressors.

### Limitations

Although the OSH Pulse data source provides several advantages, such as the use of random probability sampling which reduces the likelihood of selection bias and minimizes the impact of contextual factors, several limitations related to the measurement of key variables and the study design should be considered when interpreting the findings. *First,* both AM and health outcomes were assessed using brief, non-validated screening measures. Our operationalization of AM was based on three binary items capturing core AM functions—algorithmic monitoring, task allocation, and performance evaluation. While these items represent central features of AM [3. 4], they do not capture its full complexity, including aspects such as indirect control (e.g., gamification), dynamic pricing, or the intensity and frequency of AM use (6). Consequently, our findings should be interpreted as reflecting exposure to core AM functions rather than the broader phenomenon of algorithmic management.

Similarly, health outcomes were measured using broad indicators. Conceptually distinct conditions, such as stress, depression, and anxiety, were combined into a single outcome, and information on symptom severity, duration, or clinical diagnoses was unavailable. As a result, our analyses cannot identify specific etiological pathways but rather suggest a general association between AM exposure and adverse health. Nevertheless, the consistency of the associations across multiple health outcomes, countries, and economic sectors supports the robustness of the findings.

A similar limitation applies to the assessment of work stressors, which was also based on broad indicators. Although the items represent important occupational stressors with well-established relevance for health and therefore provide a sound basis for estimating the incremental effects of AM (16), future research should incorporate a more comprehensive range of psychosocial working conditions. Consequently, the findings of this study do not imply that the observed associations of AM persist after accounting for the full range of psychosocial working conditions. Given the documented interplay between AM and precarious forms of employment (25), as well as its potential impact on social relationships at work (24), particular attention should be paid to factors such as job insecurity and social support. Including these dimensions would further strengthen the assumption that AM is associated with additional occupational health risks.

Because both AM exposure and health outcomes were self-reported, recall bias, social desirability bias, and exposure misclassification cannot be excluded. For example, workers may not always be aware of algorithmic decision-making processes in their organization. Moreover, health problems may be underreported in the interview setting of the survey. Future research should therefore employ validated multidimensional measures of AM (11, 12), combined with organizational information about AM (e.g., job descriptions or workplace observations), and link AM exposures to clinically validated health outcomes.

*Second,* the cross-sectional design precludes causal inference. Although reverse causation cannot be ruled out entirely, it appears unlikely that existing health problems would systematically influence workers’ reports of core AM functions in their workplace. Likewise, common-method bias cannot be excluded because all variables were assessed by self-reports. However, the inclusion of a broad range of established psychosocial and occupational confounders reduces the likelihood that the observed associations are solely attributable to omitted variables or shared method variance. Nevertheless, cross-lagged panel designs using multiple data sources are desirable to establish the temporal relationship between AM exposure and health outcomes.

*Finally,* with respect to the representativeness of the sample the following should be noted: Regarding the frequency of health outcomes, it is plausible both from a neuroscientifically as well as clinically informed biobehavioral perspective that bodily complaints (headache, eyestrain) are more frequently reported than mental complaints (depression, anxiety) (26). Moreover, the observed associations between demographic variables and health outcomes are consistent with established occupational health research, including higher self-reported mental and physical health problems among women (27), and elevated injury risks among workers with lower educational attainment (28). These expected patterns support the overall plausibility of the findings. Weak or negative age effects may reflect the well-documented healthy worker effect in occupational health research (29). According to EU statistics, people with tertiary education are overrepresented in this sample (30). Given that AM has so far been mainly associated with low qualified jobs (6) the study probably underestimates the frequency of AM as well as the association between AM and health in the general working population.

### Practical implications

From an occupational safety and health (OSH) perspective, the findings underscore that AM should be considered as a relevant organizational factor in psychosocial risk assessments (31). Although observed effects were modest, the consistency of the associations across multiple health problems, countries, and sectors supports the robustness of the findings. As AM is increasingly implemented across many sectors and countries (32), even modest risks can have substantial public health implications when the exposure is widespread. Moreover, the reported odds ratios represent the incremental effect of each additional AM function. As illustrated in Figure 1, the probability of health problems increases approximately linearly with the number of AM functions to which workers are exposed. Consequently, the difference in risk between workers exposed to comprehensive AM (all three functions) and those not exposed to AM is substantially greater than the reported per-unit effect estimates suggest.

On a legislative level, the proliferation of AM necessitates targeted regulatory adaptation to mitigate emerging occupational health risks among employees. This applies in particular to the rigorous application of occupational health and safety regulations to the field of platform work, in which AM is a defining feature (33). On an organizational level, employers should particularly provide transparency of AM systems and establish formalized processes that allow employees to question and adjust AM system outputs (e.g., through access to additional contextual information), correct algorithmic decisions where appropriate, define boundaries of system functions (e.g., scope of monitoring), and clarify human responsibilities in AM augmented decision-making processes (34). Integrating such safeguards may increase workers control within AM systems and mitigate related health risks while preserving the intended efficiency gains of algorithmic systems. However, the efficacy of such approaches needs to be tested in future studies. Finally, findings are also relevant for the practice of occupational health physicians in order to assess the possible aetiology of frequent psychosomatic complaints (26).

## Data Availability

Publicly available datasets were analyzed in this study. This data can be found at: https://doi.org/10.4232/1.14208.
